# Effect of Carbon Fiber Paper with Thickness Gradient on Electromagnetic Shielding Performance of X-Band

**DOI:** 10.3390/ma17112767

**Published:** 2024-06-06

**Authors:** Zhi Liu, Meiping Song, Weiqi Liang, Xueping Gao, Bo Zhu

**Affiliations:** 1 Key Laboratory for Liquid Solid Structural Evolution and Processing of Materials (Ministry of Education), Shandong University, Jinan 250061, China; 2 Carbon Fiber Engineering Research Center, Shandong University, Jinan 250061, China; 3 School of Materials Science and Engineering, Shandong University, Jinan 250061, China

**Keywords:** carbon fiber papers, thickness gradient, electromagnetic interference shielding

## Abstract

Flexible paper-based materials play a crucial role in the field of flexible electromagnetic shielding due to their thinness and controllable shape. In this study, we employed the wet paper forming technique to prepare carbon fiber paper with a thickness gradient. The electromagnetic shielding performance of the carbon fiber paper varies with the ladder-like thickness distribution. Specifically, an increase in thickness gradient leads to higher reflectance of the carbon fiber paper. Within the X-band frequency range (8.2–12.4 GHz), reflectivity decreases as electromagnetic wave frequency increases, indicating enhanced penetration of electromagnetic waves into the interior of the carbon fiber paper. This enhancement is attributed to an increased fiber content per unit area resulting from a greater thickness gradient, which further enhances reflection loss and promotes internal multiple reflections and scattering effects, leading to increased absorption loss. Notably, at a 5 mm thickness, our carbon fiber paper exhibits an impressive average overall shielding performance, reaching 63.46 dB. Moreover, it exhibits notable air permeability and mechanical properties, thereby assuming a pivotal role in the realm of flexible wearable devices in the foreseeable future.

## 1. Introduction

With the rapid development of 5G communication, wireless power transmission, the Internet of Things, and artificial intelligence, the problem of electromagnetic pollution has followed while promoting people’s lives [1,2,3,4,5]. Electromagnetic interference (EMI) not only affects the normal operation of equipment but also potentially threatens human life and health [6,7,8]. Electromagnetic shielding materials have become a research hotspot in the fields of electronic wearable devices, national defense, aviation, aerospace, and so on.

Metal materials are limited in practical applications due to their high bulk density, poor flexibility, and easy corrosion [9,10,11,12]. Lumnitzer et al. [13] proposed measures to mitigate indoor electromagnetic fields by investigating the impact of metal materials on electromagnetic shielding efficiency. However, the utilization of metal materials in lightweight products is challenging due to their weight and applicability issues. Yim et al. [14] successfully enhanced the electromagnetic shielding performance of carbon fiber by applying a chemical metal layer on its surface; nevertheless, ensuring the environmental stability of this coating remains an unresolved concern. Flexible paper-based materials exhibit significant advantages in the future trend towards lightweight and thin structures, owing to their ability to be shaped controllably and their low bulk density. Carbon fiber (CF) is widely used in electromagnetic shielding materials [15,16,17] due to its excellent conductivity and its unique scattering effect. Hong et al. [18] employed the free space measurement method to assess the anisotropy of electromagnetic interference shielding in CFRP. Liang conducted tests on corrugated carbon fiber surface felt to achieve enhanced electromagnetic shielding performance, thereby showcasing the promising application potential of carbon-based materials in the realm of electromagnetic shielding. The paper-based carbon fiber flexible material [17,19,20], prepared through the process of paper-making using chopped carbon fibers, exhibits distinctive advantages in terms of lightweight, thinness, and wearability as electromagnetic shielding materials. In previous studies [21], we employed wet paper-making technology to fabricate cost-effective and high-performance electromagnetic shielding paper. To achieve a synergistic magnetodielectric effect for attenuating electromagnetic waves, we incorporated the dielectric loss absorbing agent MOS_2_ and magnetic loss absorbing agent Fe into the encapsulated resin. However, the introduction of metal resulted in an increased density of carbon fiber paper and susceptibility to oxidation, leading to a reduction in electromagnetic shielding efficiency.

The thickness of paper-based materials significantly influences the electromagnetic shielding properties of said material [22,23]. For instance, Huang et al. [24] prepared polydimethylsiloxane/carbon nanotube (PDMS/CNT) sandwich composites and explored the thickness-dependent microwave interference effect through the sandwich materials with different intermediate thicknesses. Gaoui et al. [25] designed a new multi-layer structural shielding layer through simulation tests, and the thickness of each layer stack gradually increased. In the frequency range of 0.8 to 1.8 GHz, simulation results show that the proposed five-layer material has very good electromagnetic interference shielding efficiency compared to the previously developed PANI 8.8/PU single-layer and three-layer materials and is a potential candidate for EMI shielding applications. With an increase in the thickness of carbon fiber paper, there is a corresponding increase in the number of carbon fibers per unit area, resulting in the construction of more conductive paths within the material and ultimately enhancing its conductivity. The electromagnetic shielding performance of the material is determined by its three-dimensional conductive network [26,27,28].

Therefore, in this study, carbon fiber paper with step-thickness variation was prepared using wet paper forming technology. The thickness of the paper plays a crucial role in its ability to respond to electromagnetic waves. This study investigates the impact of step thickness variation on the electromagnetic shielding properties of carbon fiber paper materials. Additionally, the electric heating property of carbon fiber paper enables it to possess joule heating capabilities, offering the potential for future applications in flexible heating devices. To enhance the practical performance of carbon fiber-based materials, maintaining a certain degree of air permeability while being lightweight and possessing mechanical properties is essential for carbon fiber papers with varying step thicknesses.

## 2. Materials and Methods

### 2.1. Materials

Short carbon fiber (T300) purchased from Toray Co., Ltd. (Tokyo, Japan). Polyethylene oxide solution (PEO, 0.5 wt% concentration, molecular weight 6 million) was purchased from Japan Shoyu Seika Co., Ltd. (CAS: 25322-68-3) (Tokyo, Japan). Adhesive acrylic (BR-116) resin purchased from Usolf, Ltd. (Mitsubishi Corporation, (Tokyo, Japan) CAS: 9003-01-4).

### 2.2. Experimental Scheme of Carbon Fiber Paper with Thickness Gradient

Initially, a 0.5 wt% PEO dispersion was prepared by dissolving 5 g of PEO in 1000 g of deionized water. The mixture was mechanically stirred for 30 min until significant accumulation of PEO particles ceased, followed by filtration to eliminate any impurities.

**Figure 1:**
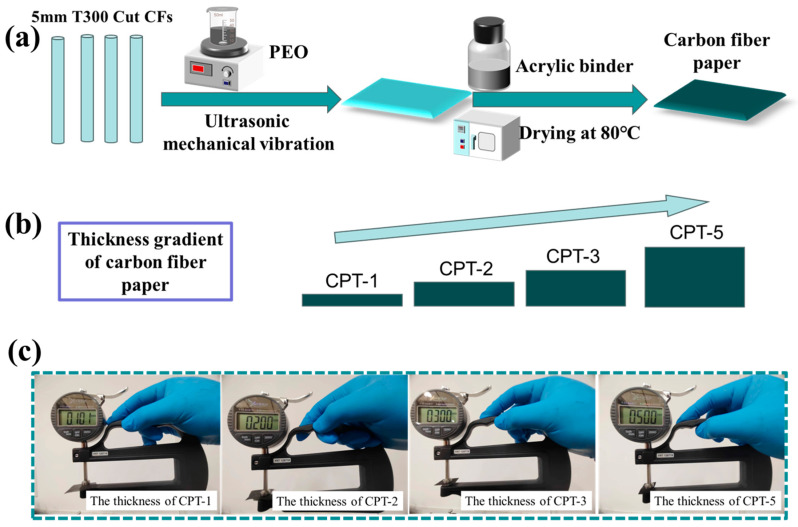
(**a**) Experimental flow chart of carbon fiber paper with thickness gradient, (**b**) carbon fiber paper with gradient thickness (The thickness showed a stepwise upward trend), and (**c**) visual picture of thickness tester testing CPT-1 to CPT-5.

Subsequently, weighed amounts (0.3 g, 0.6 g, 0.95 g, and 1.5 g) of chopped carbon fiber were added to the aforementioned PEO dispersion and subjected to ultrasonic vibration and mechanical agitation for an additional duration of 30 min until complete dispersion of the chopped fibers within the solution was achieved. Finally, the resulting dispersion underwent mold filtration, and the primary carbon fiber paper was dried using a paper dryer. The primary carbon fiber paper is impregnated with polyacrylic resin (binder) after pressing and then dried at 80 °C to prepare carbon fiber paper. The influence of paper thickness on electromagnetic shielding performance was investigated experimentally. The experimental scheme of carbon fiber paper with thickness gradient is illustrated in Figure 1a.

Carbon fiber paper with different thickness gradients in 0.1 mm, 0.2 mm, 0.3 mm, and 0.5 mm are named CPT-1, CPT-2, CPT-3, and CPT-5, respectively. Additionally, Figure 1b presents the model depicting the increase in thickness for carbon fiber paper with a gradient. The visual image of the variation in step thickness of carbon fiber paper is depicted in Figure 1c.

### 2.3. Characterization

The morphology was characterized by Hitachi su-70 scanning electron microscope (SEM, Tokyo, Japan). A thickness tester (Yuhengtong Technology Co., Ltd., (Shenzhen, China) YHT 128718) was used to test the thickness of carbon fiber paper with stepped thickness. Dc resistance tester (Hopetech Technology Co., Ltd., (Barnoldswick, UK) HK3540-3) was used to test the surface resistance of carbon fiber paper. The sample cleaning test used an ultrasonic cleaning agent (SY-180, Skymen Cleaning Equipment Shenzhen Co., Ltd. (Shenzhen, China)). According to the fabric breathability standard (ISO 9237-1995), a non-woven breathability tester (Model TQD-G1, PARAM Boper Co., Ltd. (Wales, UK)) was used to measure the breathability of the sample.

The scattering parameters (*S*_11_ and *S*_12_ or *S*_21_ and *S*_22_), the relative complex permittivity ($\varepsilon_{r}=\varepsilon^{\prime }-j\varepsilon^{\prime \prime }$) and the complex permeability ($\mu_{r}=\mu^{\prime }-j\mu^{\prime \prime }$) were obtained by Agilent PNA-N5244A vector network analyzer (Agilent Technologies, Inc., Palo Alto, CA, USA) frequency range 8.2–12.4 GHz (X-band) was measured using waveguide method. All custom paper samples (22.86 mm × 10.16 mm) with copper sample clamp and the connection between the waveguide flanges. The reflection coefficient (*R*) and transmission coefficient (*T*) were determined by the *S* parameters (*S*_11_, *S*_12_, *S*_21_, *S*_22_). There was an equilibrium between *R*, *T*, and the absorption coefficient (*A*), which could be calculated using the following formula [29,30,31]:

(1) $$R=|S_{11}{|}^{2}=|S_{22}{|}^{2}$$

(2) $$T=|S_{12}{|}^{2}=|S_{21}{|}^{2}$$

*R* + *T* + *A* = 1 (3)

According to Schelkunoff’s theory, the total EMI SE (*SE*_T_) was the sum of surface reflection loss (*SE*_R_), multiple reflection loss (*SE*_M_), and absorption loss (*SE*_A_) inside the material, and could be expressed in the following equation [27,32,33,34]:

(4) $$SE_{T}\left(dB\right)=-10\log\left(\frac{P_{T}}{P_{I}}\right)=SE_{R}+SE_{M}+SE_{A}$$

(5) $$SE_{R}\left(dB\right)=-10\log\left(1-S_{11}^{2}\right)=-10\log\left(1-S_{22}^{2}\right)$$

(6) $$SE_{A}\left(dB\right)=-10\log\left(\frac{S_{12}^{2}}{1-S_{11}^{2}}\right)=-10\log\left(\frac{S_{21}^{2}}{1-S_{11}^{2}}\right)$$

There are studies that show that the *SE*_M_ could be neglected when the *SE*_T_ > 15 dB and it could be described as [5,35,36,37,38]:

(7) $$SE_{T}\approx SE_{R}+SE_{A}$$

## 3. Results and Discussion

### 3.1. Microstructure and Light Transmittance Analysis

**Figure 2:**
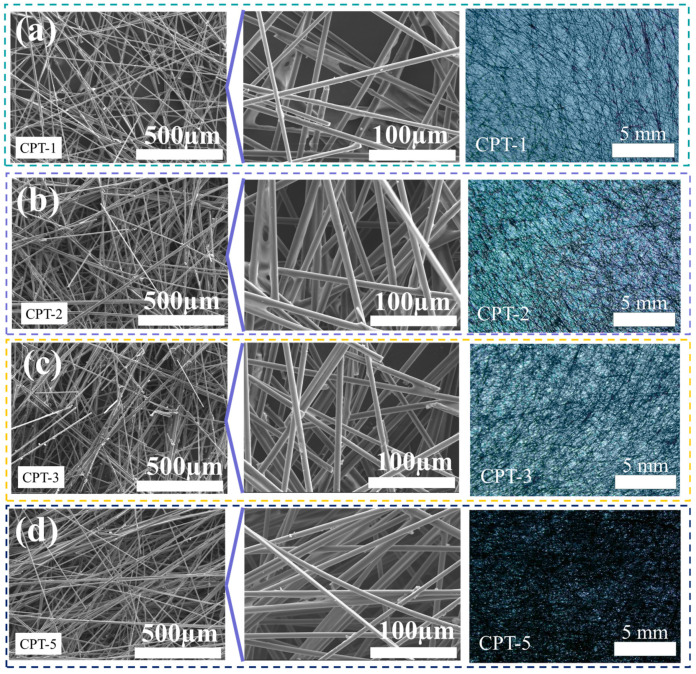
SEM image and light transmission analysis of (**a**) CPT-1, (**b**) CPT-2, (**c**) CPT-3, and (**d**) CPT-5 step-thickness carbon fiber paper.

The SEM topography and light transmittance of carbon fiber paper CPT-1 to CPT-5 are presented in Figure 2. The scanning electron microscopy (SEM) analysis revealed that the short-cut carbon fibers were randomly interlaced to form a matrix in the carbon fiber paper. As the gradient thickness of the carbon fiber paper increased, there was an observed increase in the carbon fiber content per unit area within the paper substrate. In the context of wearable devices, carbon fiber paper exhibits commendable breathability. Figure S1a illustrates the breathability characteristics of carbon fiber paper materials. As the ladder thickness increases, there is a corresponding decrease in air permeability due to an augmented number of fibers per unit area, resulting in reduced material permeability. Under a pressure of 2.5 Pa, CPT-1 to CPT-5 exhibit air permeabilities of 25.47 L/min, 9.72 L/min, 5.99 L/min, and 2.95 L/min, respectively.

The compactness of the carbon fiber paper was assessed through its light transmittance, which varies with different thicknesses. As the thickness increases, there is an increase in fiber content per unit area and a decrease in light transmittance for the carbon fiber paper samples. Notably, when reaching a thickness of 0.5 mm, CPT-5 exhibits weak surface light transmission with minimal white light transmission area and relatively high density of surface fibers. In order to fulfill the lightweight performance requirements of paper-based materials, Figure S1b illustrates the calculated surface density of carbon fiber paper. The variation in step thickness of carbon fiber paper leads to a corresponding change in its surface density. Evidently, an increase in the thickness of carbon fiber paper results in a higher fiber content per unit area and subsequently raises the surface density. Specifically, CPT-1 exhibits a surface density of 7.77 g/m^2^, while CPT-5 reaches 49.13 g/m^2^. Similarly, CPT-2 and CPT-3 possess surface densities of 11.47 g/m^2^ and 30.47 g/m^2,^ respectively.

### 3.2. Electrical Properties and Electromagnetic Shielding Properties

**Figure 3:**
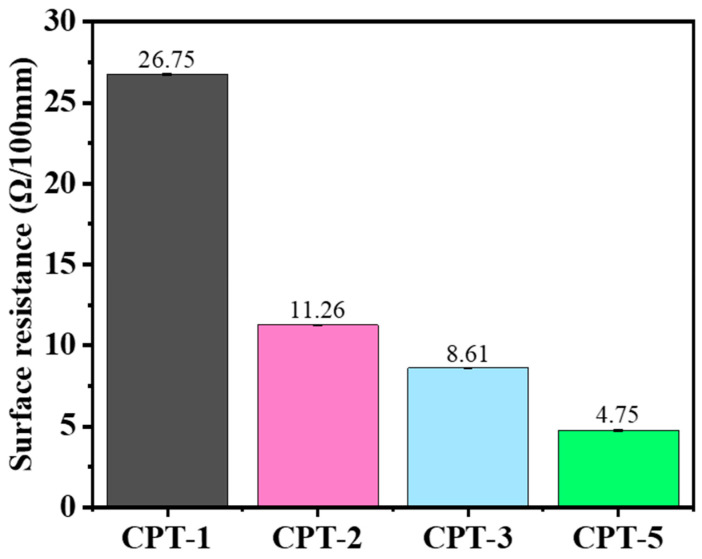
Surface resistance of carbon fiber paper. (To facilitate the distinction, the color of CPT-1 is black, the color of CPT-2 is red, the color of CPT-3 is blue, and the color of CPT-5 is green).

The electromagnetic shielding performance of the material is closely related to its surface conductivity, which in turn directly affects its reflectivity. The surface resistance of carbon fiber paper with stepped thickness variation within 100 mm was measured using a surface resistance tester, and the corresponding results are presented in Figure 3. The results demonstrate that the surface conductivity of carbon fiber paper increases proportionally with the increase in carbon fiber content per unit area. Consequently, there is a corresponding decrease in the surface resistance of carbon fiber paper. Specifically, the average surface resistance for CPT-1 measures at 26.75 Ω/100 mm, while due to enhanced electrical conductivity, this value drops significantly to 4.75 Ω/100 mm for CPT-5 carbon fiber paper. This phenomenon directly influences the reflection behavior of electromagnetic waves on the surface of carbon fiber paper.

**Figure 4:**
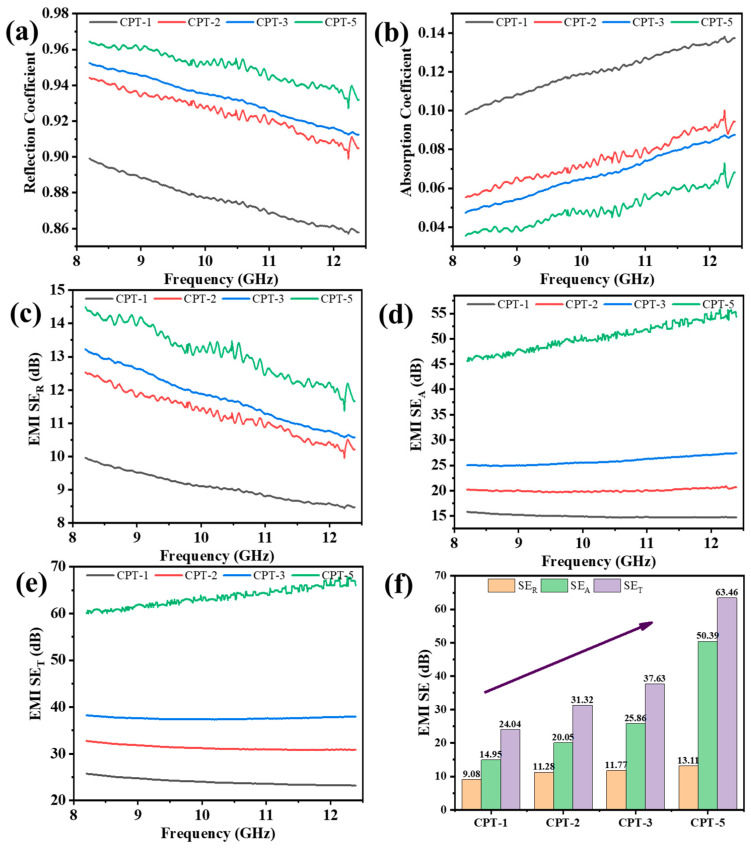
Carbon fiber paper in the X-band (**a**) reflection coefficient *R*, (**b**) absorption coefficient *A*, (**c**) reflection loss *SE*_R_, (**d**) absorption loss *SE*_A_, (**e**) overall shielding performance *SE*_T_, and (**f**) average EMI *SE* of the carbon fiber paper material (Arrows show an overall upward trend).

The electromagnetic parameters of carbon fiber paper in the X-band were tested by the waveguide cavity, and the test results are shown in Figure 4. Figure 4a shows the reflection coefficient R curve of carbon fiber paper. The reflection coefficient of the whole carbon fiber paper is above 0.86, and the average R-value from CPT-1 to CPT-5 is 0.876, 0.925, 0.932, and 0.95, respectively. This shows that when the electromagnetic wave reaches the surface of the carbon fiber, nearly 90% of the electromagnetic wave is effectively reflected, and a large number of electromagnetic waves are shielded.

With the increase in thickness, the reflection coefficient is further enhanced. Through light transmission, it can also be analyzed that the fiber content per unit area increases with the increase in thickness, the surface conductive network is enhanced, and the reflection ability of carbon fiber paper is strengthened. In the X-band, with the increase in electromagnetic wave frequency, the R curve of carbon fiber paper shows a downward trend as a whole; because the electromagnetic wave frequency increases, more electromagnetic waves enter the carbon fiber paper, and then the reflection coefficient curve of the material shows an overall downward trend. The results of the absorption coefficient A curve of carbon fiber paper are shown in Figure 4b. In the X-band range, with the increase in thickness, the absorption coefficient of carbon fiber paper is opposite to the reflection coefficient, showing a downward trend. Similarly, with the increase in electromagnetic wave frequency, the absorption coefficient A is opposite to the reflection coefficient, and the overall trend is rising.

The shielding efficiency of carbon fiber paper is calculated according to formulas 4 to 6. The results are shown in the reflection loss *SE*_R_ (Figure 4c), absorption loss SE_A_ (Figure 4d), and the overall shielding performance *SE*_T_ (Figure 4d) of carbon fiber paper material. The increase in step thickness leads to an augmented reflection loss and absorption loss of carbon fiber paper, thereby further enhancing the overall electromagnetic shielding performance. As the thickness gradient increases in the carbon fiber paper, resulting in an augmented content of carbon fibers per unit area and an enhanced three-dimensional conductive network effect, reflection loss occurs due to interactions between surface-free electrons and the incident electromagnetic wave. The interior-bound electromagnetic waves undergo multiple reflections and scattering effects, leading to their conversion into heat through electric dipole polarization. The increase in thickness enhances the multiple reflection and scattering effects of electromagnetic waves inside the carbon fiber paper, which leads to an upward trend in the reflection loss and absorption loss curves. Alterations in the thickness gradient modify the transmission path of electromagnetic waves within the carbon fiber paper, consequently influencing its absorption loss. Electromagnetic waves penetrating the interior of carbon fiber interact with its three-dimensional conductive network structure, causing surface electrons to convert electromagnetic energy into thermal energy. The variation in step thickness results in an increased multi-layer interface of carbon fiber paper, thereby enhancing the interface polarization effect. Simultaneously, the unique destructive interference effect of carbon fiber further contributes to electromagnetic wave attenuation. Consequently, the absorption loss of carbon fiber paper is significantly amplified, leading to an overall improvement in the material’s electromagnetic shielding performance.

The average electromagnetic loss performance parameters of carbon fiber paper in the X-band were further calculated, and the results are shown in Figure 4f. As shown in the figure, the absorption loss is greatly enhanced with the further increase in reaction thickness, from 14.95 dB of CPT-1 material to 50.39 dB of CPT-5 material. The electromagnetic shielding efficiency, which is mainly based on absorption loss, is also increased from 24.04 dB of CPT-1 to 63.46 dB of CPT-5.

**Figure 5:**
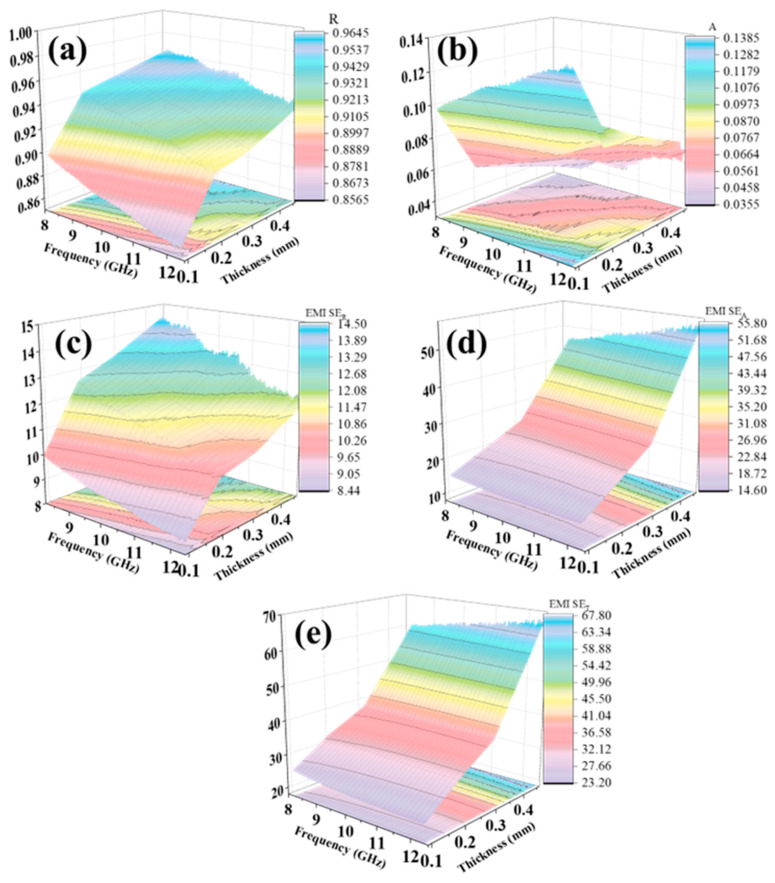
3D plot of electromagnetic parameters (**a**) *R*, (**b**) *A*, (**c**) *SE*_R_, (**d**) *SE*_A_, and (**e**) *SE*_T_ in relation to thickness at X-band.

In order to more accurately depict the relationship between electromagnetic parameters, thickness, and frequency in the X-band, a corresponding 3D diagram is presented in Figure 5. The figure intuitively illustrates that changes in carbon fiber paper thickness result in an increase in the reflection coefficient (Figure 5a) and a decrease in the absorption coefficient (Figure 5b) within the X-band. Additionally, an increase in carbon fiber content per unit area enhances conductive network effects, leading to improved electromagnetic performance reflected by increased reflection loss *SE*_R_ (Figure 5c) and absorption loss *SE*_A_ (Figure 5d), ultimately improving overall electromagnetic shielding performance *SE*_T_ as shown in Figure 5e.

### 3.3. Joule Heating Properties

**Figure 6:**
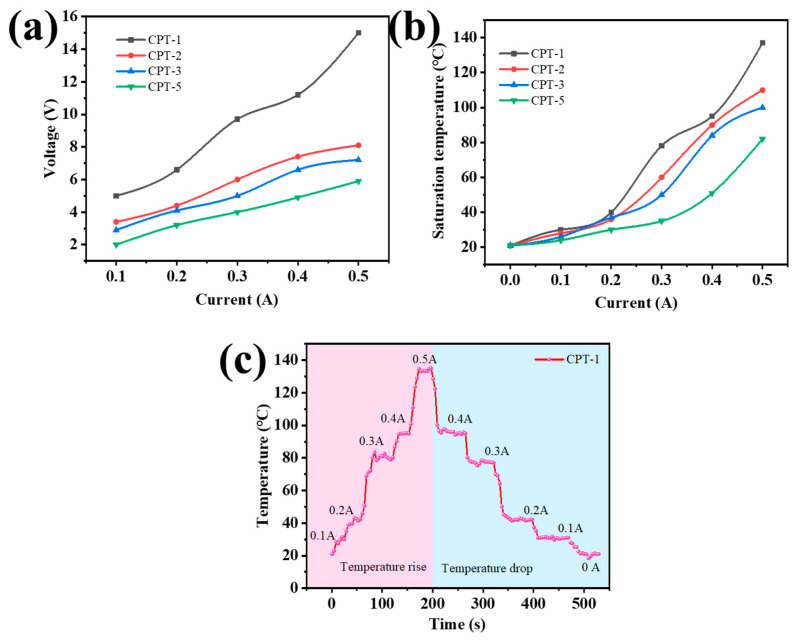
(**a**) Voltage–current change curves, (**b**) saturation temperature of carbon fiber paper with different current thickness gradients, and (**c**) CPT-1 carbon fiber paper under different current temperature change curves.

The joule heating properties of carbon fiber paper have significant applications in wearable heaters [39,40,41,42]. To investigate the Joule heat change, the voltage was controlled using a DC power supply while the temperature change was measured using an infrared imaging recorder. In Figure S2, infrared temperature maps were recorded to observe gradient changes in carbon fiber paper. The surface resistance of carbon fiber paper decreases with increasing thickness, enabling CPT-1 to achieve high-temperature Joule heat at low current levels. The heat-conducting network of carbon fiber makes the material respond quickly to temperature. The current–voltage curve (Figure 6a) further confirms the surface resistance of carbon fiber paper, with CPT-1 exhibiting relatively higher surface resistance and voltage values compared to other materials under the same current conditions. At a current of 0.5 V, CPT-1 reaches a voltage of 15 V, while CPT-2 to CPT-5 exhibit voltages of 8.1 V, 7.2 V, and 5.9 V, respectively. In Figure 6b, maximum saturation temperatures are shown for different currents applied to CPT-x. The maximum saturation temperature achieved by CPT-1 is higher than that of other materials at various currents due to its lower surface resistance. Joule heat is directly related to surface resistance. In just 30 s at a current of 0.5 A, CPT-1 reaches a maximum saturation temperature as high as 137 °C; CPT-2 achieves 110 °C; CPT-3 attains 100 °C, and finally, CPT-5 records 82 °C. Figure 6c demonstrates how controlling the current allows for precise control over the temperature change in CPT-1. These unique joule thermal properties make it an ideal choice for wearables and heating components.

### 3.4. Practical Application Performance of Carbon Fiber Paper

**Figure 7:**
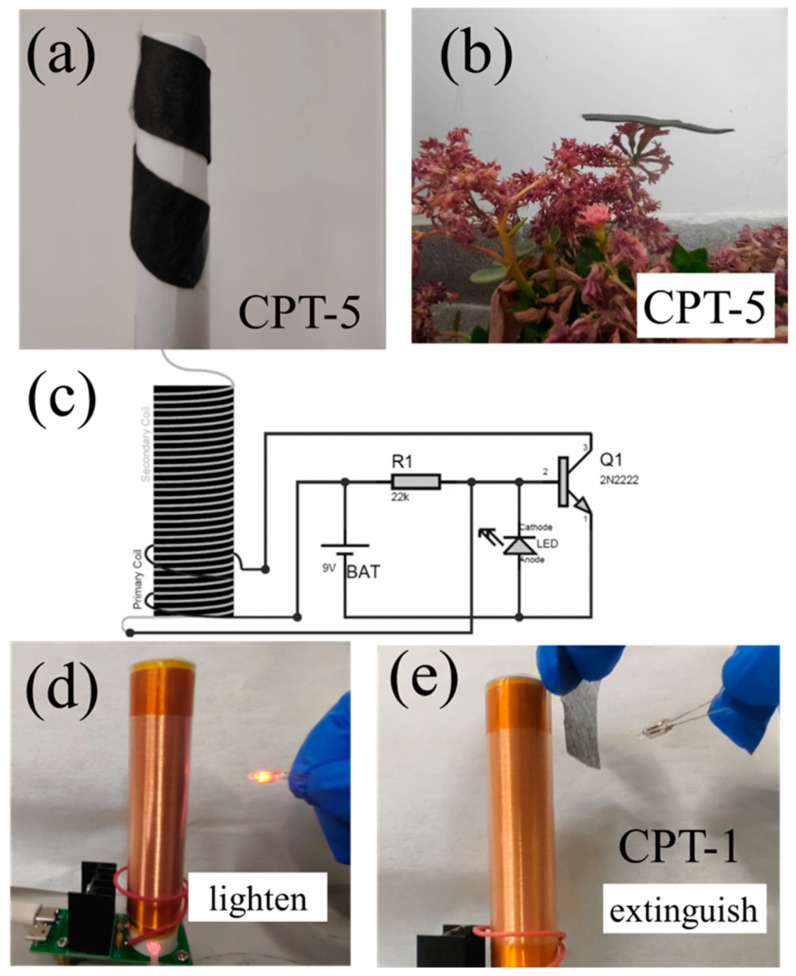
CPT-5 is (**a**) wound on the surface of a cylindrical barrel and (**b**) placed on the stamen; (**c**) Schematic diagram of Tesla coil; (**d**) The Tesla coil is lit in space. (**e**) CPT-1 blocks electromagnetic wave propagation and the small bulb is extinguished.

Carbon fiber paper, renowned for its lightweight and flexible properties, finds significant applications as an electromagnetic shielding material. As shown in Figure 7a, CPT-5 (20 mm × 70 mm) can be easily wound around a cylindrical paper tube. At the same time, it is shown in Figure S3 that other CPT-x materials are also flexible. In addition to the surface density in Figure S1b, Figure 7b illustrates the placement of CPT-5 (50 mm × 50 mm) on stamens without notable deformation, emphasizing its lightweight and flexible nature. Similarly, Figure S4 also reflects the lightweight characteristics of other CPT-x materials in practical application scenarios, as depicted in the picture placed on the flower core.

The Tesla coil operates by converting AC power supply through a transformer and utilizing a shunt capacitor to continuously raise the voltage and generate high-frequency electromagnetic waves. Figure 7c provides a schematic representation of its working principle. In Figure 7d, electromagnetic waves generated by the Tesla coil light the bulb in space. By positioning CPT-1 between the small light bulb and the Tesla coil, we observed that the electromagnetic shielding effect of CPT-1 effectively blocked the propagation of electromagnetic waves, resulting in the extinguishing of the light bulb. This validates the electromagnetic shielding capabilities of CPT-1. Figure S5 shows that other CPT-x also have the characteristics of blocking electromagnetic waves in practical applications.

## 4. Conclusions

In summary, carbon fiber paper with stepped thickness was fabricated using wet paper forming technology. The electromagnetic response effects of the carbon fiber paper with stepped thickness were found to vary. The 0.1 mm carbon fiber paper exhibits an electromagnetic loss performance of 24.04 dB. As the thickness of the carbon fiber paper increased, the 0.5 mm thick sample exhibited a shielding performance of 63.46 dB in the X-band frequency range. Furthermore, it was observed that carbon fiber paper possesses certain joule heating capabilities, achieving a rapid thermal response of 137 °C within just 30 s at a low current of 0.5 A. Additionally, carbon fiber paper exhibits air permeability and light surface density while maintaining satisfactory flexible properties, making it suitable for practical applications as wearable-based materials. The utilization of carbon fiber paper as a prospective electromagnetic shielding material in the future is anticipated, showcasing its inherent advantages within flexible substrate materials.

## Data Availability

The raw data supporting the conclusions of this article will be made available by the authors on request.

## Supplementary Materials

The following supporting information can be downloaded at https://www.mdpi.com/article/10.3390/ma17112767/s1, Figure S1: Carbon fiber paper (a) permeability and (b) surface density; Figure S2: Infrared imaging of temperature changes of carbon fiber paper under different current currents; Figure S3: CPT-x is easily wound on the surface of a cylindrical cylinder, (a) CPT-1, (b) CPT-2, (c) CPT-3; Figure S4: CPT-x stands on the flower, (a) CRT-1, (b) CRT-2, (c) CRT-3; Figure S5: CPT-x blocks electromagnetic waves from the Tesla coil, (a) CRT-2, (b) CRT-3, (c) CRT-5.
