# Supplementary material for: Effect of Carbon Fiber Paper with Thickness Gradient on Electromagnetic Shielding Performance of X-Band
Source: Materials (Basel). 2024 Jun 6;17(11):2767. doi: 10.3390/ma17112767 (PMC11173914; doi:10.3390/ma17112767)
Supplement: Supplementary file 1 [file materials-17-02767-s001.zip › materials-2976521-supplementary.pdf]

Effect of carbon fiber paper with thickness gradient on electromagnetic shielding performance of X-band

Zhi Liu[a,b] , Meiping Song[a,b], Weiqi Liang[c], Xueping Gao[a,b]* , Bo Zhu [a,b]**

[a]Key Laboratory for Liquid Solid Structural Evolution and Processing of Materials (Ministry of Education), School of Materials Science and Engineering, Shandong University, Jinan 250061, China

[b]Carbon Fiber Engineering Research Center, School of Material Science and Engineering, Shandong University, Jinan 250061, China

[c]School of Materials Science and Engineering, Shandong University, Jinan 250061, PR China

The air permeability of carbon fiber paper with stepped thickness and the surface density of carbon fiber paper are evaluated in Fig.S1. It is observed that an increase in the thickness of the characterization material leads to a decrease in its permeability. Consequently, wearable materials should be selected based on their corresponding permeability requirements. Additionally, the surface density serves as an indicator for the lightweight properties of carbon fiber paper.

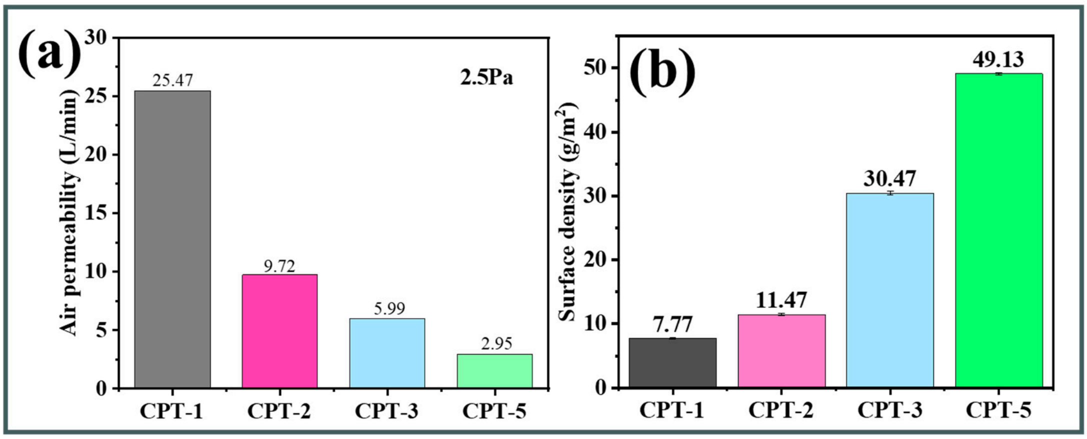

Figure S1. Carbon fiber paper (a) permeability and (b) surface density

The infrared imager was employed to capture stable infrared images of carbon fiber paper CPT-x under varying current conditions.

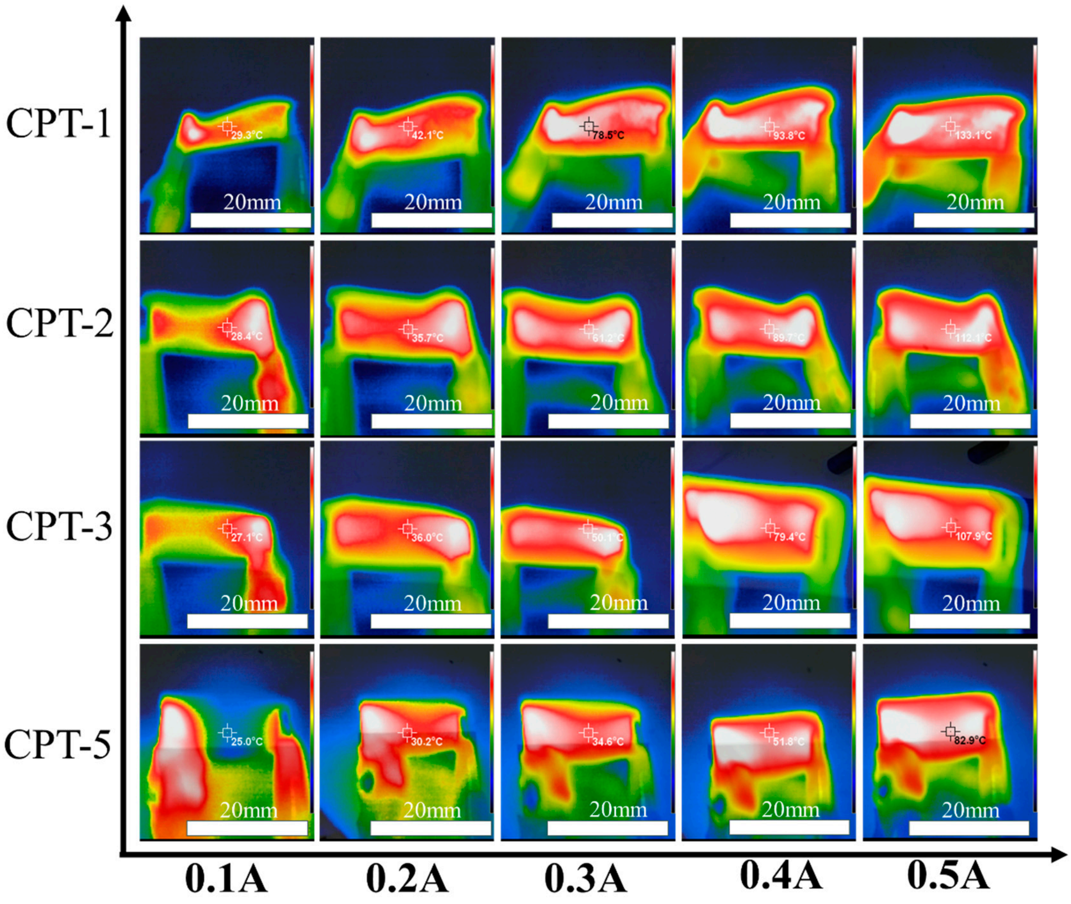

Figure S2. Infrared imaging of temperature changes of carbon fiber paper under different current currents

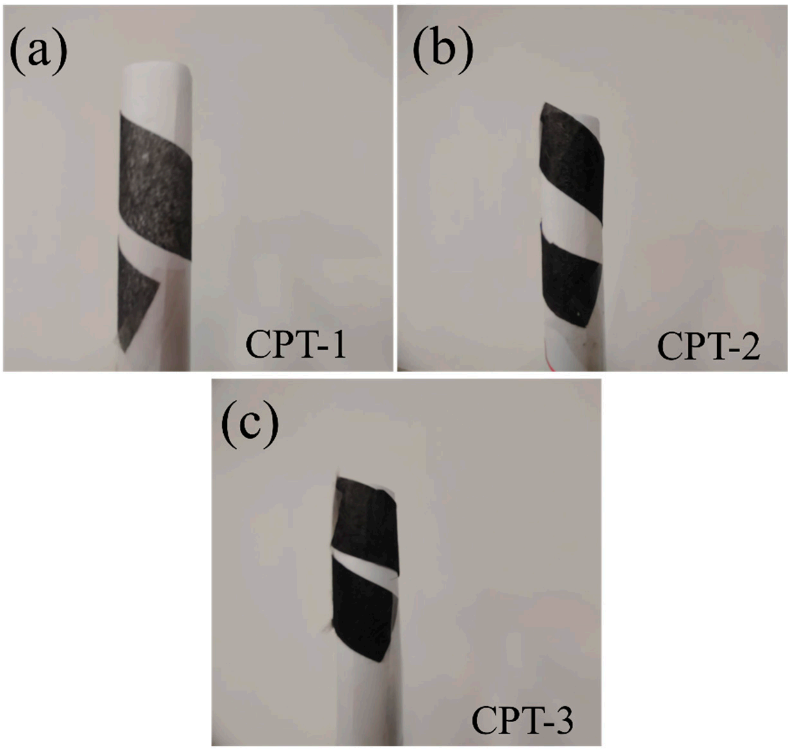

Figure S3. CPT-x is easily wound on the surface of a cylindrical cylinder, (a)CPT-1, (b)CPT-2, (c)CPT-3

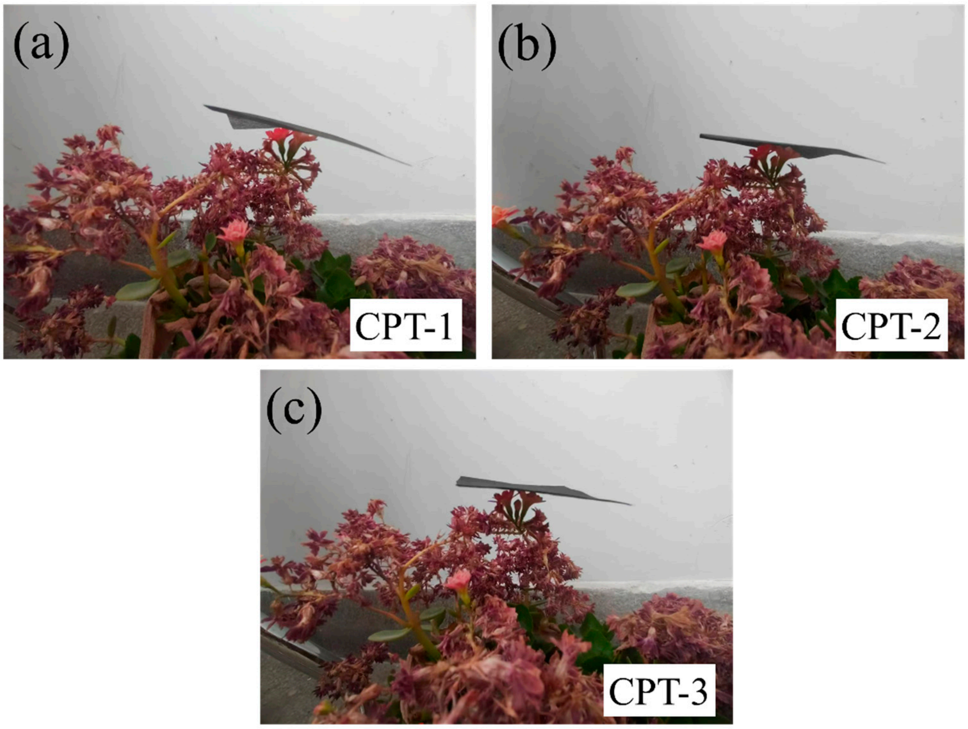

Figure S4. CPT-x stands on the flower，(a)CRT-1, (b)CRT-2, (c)CRT-3

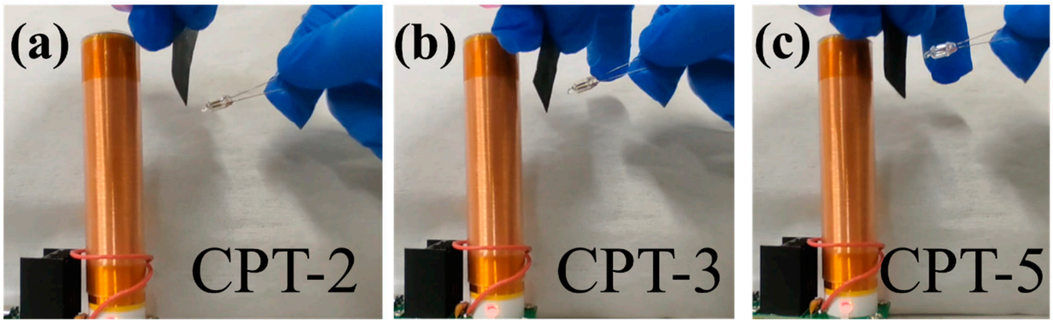

Figure S5. CPT-x blocks electromagnetic waves from the Tesla coil
